# Raman spectroscopy enables phenotyping and assessment of nutrition values of plants: a review

**DOI:** 10.1186/s13007-021-00781-y

**Published:** 2021-07-15

**Authors:** William Z. Payne, Dmitry Kurouski

**Affiliations:** 1 Department of Biochemistry and Biophysics, Texas A&M University, College Station, TX 77843 USA; 2 Department of Biomedical Engineering, Texas A&M University, College Station, TX 77843 USA

**Keywords:** Digital farming, Raman spectroscopy, Phenotyping, Nutrition value identification

## Abstract

Our civilization has to enhance food production to feed world’s expected population of 9.7 billion by 2050. These food demands can be met by implementation of innovative technologies in agriculture. This transformative agricultural concept, also known as digital farming, aims to maximize the crop yield without an increase in the field footprint while simultaneously minimizing environmental impact of farming. There is a growing body of evidence that Raman spectroscopy, a non-invasive, non-destructive, and laser-based analytical approach, can be used to: (i) detect plant diseases, (ii) abiotic stresses, and (iii) enable label-free phenotyping and digital selection of plants in breeding programs. In this review, we critically discuss the most recent reports on the use of Raman spectroscopy for confirmatory identification of plant species and their varieties, as well as Raman-based analysis of the nutrition value of seeds. We show that high selectivity and specificity of Raman makes this technique ideal for optical surveillance of fields, which can be used to improve agriculture around the world. We also discuss potential advances in synergetic use of RS and already established imaging and molecular techniques. This combinatorial approach can be used to reduce associated time and cost, as well as enhance the accuracy of diagnostics of biotic and abiotic stresses.

## Introduction

Malnutrition due to a lack of nutritious food is an issue that over a billion people around the world face daily [1]. This problem can be solved by continuous expansion of farm land or by development of innovative agricultural approaches. One can envision that the first strategy is destructive and unlikely can be inefficient in the long term. The alternative strategy is focused on the enhancement of farming efficiency. This innovative agricultural philosophy is known as digital farming or precision agriculture. Digital Farming aims to develop innovative technological approaches that can be used to maximize the crop yield with minimal environmental impact [2, 3]. Efficient digital farming requires sensing methods that can deliver information about the plant health from a field to the farmer. Sensing methods are also essential for plant breeding. Currently, plant crosses are performed by visual analysis of plants, as well as by laboratory-based analysis of their nutrients (micro and macro elements) and nutritional values (levels of protein, starch, fiber, etc.). Such analyses require labor and time-consuming wet-laboratory techniques, such as Dumas combustion method [4] and Megazyme Total Starch Content assay (subsequently megazyme assay) [5]. This substantially decelerates the speed and confidence level of plant breeding. Timely access to the information about plant health allows for detection and identification of pests and plant diseases in the field. Such information can be used for a precise and site-specific administration of the chemical treatment that could prevent the spread of such biotic stresses and save up to 30% of the crop yield [6, 7]. Crop losses from abiotic stresses, such as drought and nutrient deficiency, are far more significant and can reach up to 70% worldwide [6, 7]. One can expect that timely sensing of plant deficiency in macro and micro elements can be used for site-specific spread of fertilizers in the field. Timely provided nutrients will mitigate the decrease in the crop yield. Moreover, such a dose-dependent administration of fertilizers can be done on the level of individual plants [8]. This will also minimize the health and environmental impacts of pollution from fertilizers.

An alternative strategy to address the issue with the crop yield losses due to drought and salinity stress can involve a development of the germplasm that has higher drought or soil salinity tolerance. However, conventional phenotyping techniques are time and labor consuming [9, 10]. Some of the currently used biophysical approaches are capable of probing physiological changes or plant chlorophyll contents. However, information provided by those methods are not directly related to stress response and so require many experiments to draw useful conclusions. The alternative biochemical approaches are more relevant but are extremely laborious [11–13]. It should be noted that experimental conditions in field experiments are very difficult to control, which further complicates elucidation of potential plant resistance to biotic and abiotic stresses that has to be determined upon plant crossings [14]. Therefore, there is a strong demand for the robust phenotyping techniques that could be used for non-destructive, accurate, and rapid assessment of breeding populations for drought related responses, especially at early seedling stages with short periods of withholding water. Such techniques ideally should identify biomarkers associated with drought resistance, as well as biochemical changes in plants associated with drought. One can expect that methodology that will enable identification of drought stress on very early (pre-symptomatic) stages could be used to differentiate between drought resistant and susceptible plants with high accuracy. This catalyzes the search for a non-invasive, non-destructive, portable, and confirmatory approaches that could be used for on-site assessment of nutrients and nutritional values of live plants and their seeds.

Recently reported research findings show that Raman spectroscopy (RS) can be used for diagnostics of biotic and abiotic stresses [15–18]. RS is a label-free laser-based technique that requires no chemicals for analysis of the plant material. This lowers the reagent cost-per-analysis value of such tests to zero [19], whereas the cost of alternative molecular methods of analysis remain high (PCR and ELISA analyses are around $25 and $13 per sample, respectively). Moreover, it takes only one second to perform the analysis of a plant to detect the presence of pathogens or reveal the origin of abiotic stresses. Considering the portable nature of RS, one can expect that ultimately Raman spectrometers will be installed on combines and grain elevators, as well as on unmanned aerial vehicle (UAVs) enabling continuous surveillance of agricultural territories. We also expect that RS can be used in concert with molecular methods of analyses. In this case, RS can be used for quick screening of the plant health; if more accurate identification of the pathogen is needed, qPCR, PCR or ELISA can be used.

When the sample is illuminated by electromagnetic radiation, the vast majority of photons that scatter back will have the same energy as the incident photons. An Indian scientist, C. V. Raman experimentally demonstrated that a very small fraction of photons (only one photon in 10 million) that hit the sample will scatter back with a different energy. These inelastically scattered photons interacted with molecules in the sample. As a result, the molecules were advanced to higher energy states. Next, molecules relax to a vibrational energy level that is different from the original molecular energy state. As a result, photons with a different (higher or lower) than the incident photon are produced. The difference in energy between the incident and inelastically scattered photon is the called Raman shift.

**Fig. 1:**
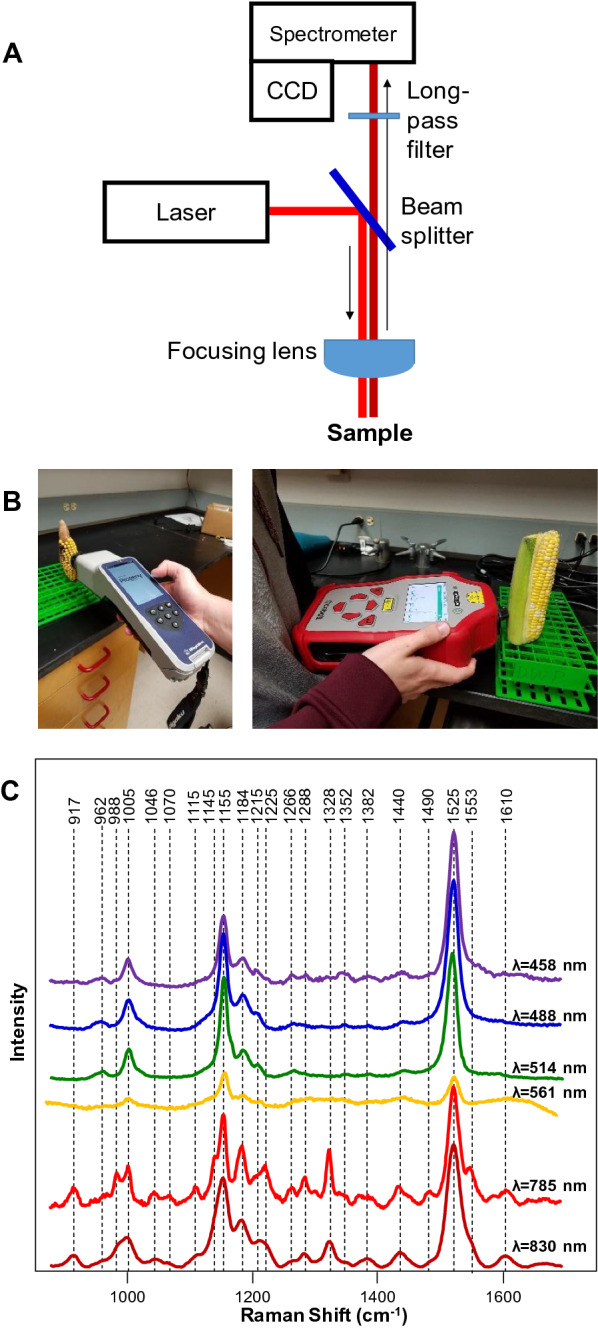
Schematic representation of a Raman spectrometer (**A**); commercially available hand-held Raman spectrometers with 1064 nm (left) and 830 nm (right) excitations (**B**). Raman spectra collected from a rose leaf with 458, 488, 514, 561, 785 and 830 nm excitations (**C**)

A Raman spectrometer is composed of a laser source that is used to generate a photon flux. Next, the light is directed by a beam splitter and is focused by a lens onto the sample, Fig. 1A. Scattered light is collected typically using the same optics and directed into the spectrometer. Prior to entering the spectrometer, elastically scattered photons are cut off using long-pass filters. After in-elastically scattered photons (Raman photons) are dispersed on the spectrometer gratings according to their energies, they are captured using CCDs.

Although RS was implemented first as a bench-top technique, currently, there are several commercially available hand-held spectrometers, Fig. 1B. These instruments typically have laser excitation in the green (λ = 532 nm), red (λ = 785 or 830 nm) or infrared (λ = 1064 nm) parts of the electromagnetic spectrum [15–17, 20–22], Table 1. Beam diameter or laser spot size on such devises range from several dozens of microns to a few millimeters. This is an important instrumental parameter that has to be taken into consideration for spectroscopic analysis of biological specimens. Plants are highly heterogeneous from perspective of their structure and composition. Hand-held spectrometers with ~ 25 microns’ beam spot, such as Rigaku Progeny, offer high spatial resolution that can be used to investigate the structural fine elements of plant leaves, such as veins and midribs. Also, an analysis with a small laser spot size requires substantially less amount of material. At the same time, if high throughput in sample analysis is necessary, the small laser spot may become disadvantageous because it will require more precise beam focusing on the plant surface. In such cases, the use of large beam diameters (a few millimeters) that are offered by Agilent Resolve can be preferred.

**Table 1:** Summary table of reported to date Raman studies on botanicals

Target	Objective	Instrumentation/parameters	Peaks with increase in intensity	Peaks with decrease in intensity	Conclusion
Disease diagnostics					
Tomato, leaf	Liberibacter disease in tomatoes [8]	Handheld spectrometer (λ = 830 nm; P = 495 mW; T = 1 s)	–	747 cm^−1^ (pectin); 1000, 1115, 1155, 1184, 1218 and 1525 cm^−1^ (carotenoids)	Liberibacter disease in tomatoes is associated with degradation and fragmentation of host carotenoids and pectin
Orange and grapefruit, leaves	Huanglongbing (HLB) or citrus greening [23]	Handheld spectrometer (λ = 830 nm; P = 495 mW; T = 1 s)	1601–1630 cm^−1^ (phenylpropanoids; 1440–1455 cm^−1^ (aliphatic)	1184 and 1218 cm^−1^ (xylan, carotenoids); 1525 cm^−1^ (carotenoids), as well as 1288 cm^−1^ (aliphatic); 1155 and 1326 cm^−1^ (cellulose)	HLB is associated with an increase in phenylpropanoids and decrease in xylan, carotenoids and cellulose
Orange and grapefruit, leaves	Nutrient deficiency in citrus trees [23]	Handheld spectrometer (λ = 830 nm; P = 495 mW; T = 1 s)	1247, 1601–1630 cm^−1^ (phenylpropanoids; 1440–1455 cm^−1^ (aliphatic)	1184 and 1218 cm^−1^ (xylan, carotenoids)	ND is associated with an increase in phenylpropanoids
Orange, leaf	Canker [22]	Handheld spectrometer (λ = 830 nm; P = 495 mW; T = 1 s)	–	1601–1630 cm^−1^ (phenylpropanoids)	Canker is associated with a decrease in phenylpropanoids content
Orange, leaf	HLB and blight [22]	Handheld spectrometer (λ = 830 nm; P = 495 mW; T = 1 s)	–	–	Diagnostics was achieved via the use of PLS-DA
Wheat, grain	Ergot [15]	Handheld spectrometer (λ = 1064 nm; P = 200 mW; T = 30 s)	1650 and 1667 cm^−1^ (proteins)	–	ergot infection may be associated with expression and deposition of alpha-helical and beta-sheet proteins
Wheat, grain	Black tip [15]	Handheld spectrometer (λ = 1064 nm; P = 200 mW; T = 30 s)	1348 cm^−1^ (monomeric sugars) and 1600 cm^−1^ (lignin); shift of 862 peak to 856 cm^−1^ (pectin)	862 and 937 cm^−1^ (starch)	black tip may degrade lignin and ferment starch into monomeric sugars; esterification of pectin
Sorghum, grain	Mold [15]	Handheld spectrometer (λ = 1064 nm; P = 200 mW; T = 30 s)	shift of 856 peak to 862 cm^−1^ (pectin); change in ratio between 1518 cm^−1^ and 1541 cm^−1^ peaks (carotenoids)	1600 and 1630 cm^−1^ (phenylpropanoids)	Degradation of phenylpropanoids; a decrease in methylesterfication of pectin caused by the infections; suggest a decrease in the length of conjugated double bonds of carotenoids
Sorghum, grain	Ergot [15]	Handheld spectrometer (λ = 1064 nm; P = 200 mW; T = 30 s)	1150, 940, 1124 and 1083 cm^−1^ (monomeric sugars); shift of 856 peak to 862 cm^−1^ (pectin); change in ratio between 1518 cm^−1^ and 1541 cm^−1^ peaks (carotenoids)	1600 and 1630 cm^−1^ (phenylpropanoids)	ergot hydrolyzes starches to produce monomeric sugars; a decrease in methylesterfication of pectin caused by the infections; suggest a decrease in the length of conjugated double bonds of carotene
Maize, grain	*Fusarium spp* [16]	Handheld spectrometer (λ = 1064 nm; P = 200 mW; T = 30 s)	1658 cm^−1^ (protein); 1153 cm^−1^ (starch)	1600 and 1633 cm^−1^ (phenylpropanoids); 1547 cm^−1^ (shifted from 1523 cm^−1^ in healthy) species (carotenoids)	*Fusarium infection is associated with degradation of* phenylpropanoids and deposition of protein in maize kernels; pathogen converts monomeric sugars polymeric carbohydrates
Maize, grain	*Aspergillus flavus* [16]	Handheld spectrometer (λ = 1064 nm; P = 200 mW; T = 30 s)	1003–1115 cm^−1^ (monomeric sugars); 1600–1633 (phenylpropanoids)	1600 and 1633 cm^−1^ (phenylpropanoids); 1547 cm^−1^ (shifted from 1523 cm^−1^ in healthy) species (carotenoids); 1153 cm^−1^ (starch)	A. flavus is associated with a breakdown maize starch into monomeric sugars
Maize, grain	*A. niger* [16]	Handheld spectrometer (λ = 1064 nm; P = 200 mW; T = 30 s)	1153 cm^−1^ (starch); 1600–1633 (phenylpropanoids)	1600 and 1633 cm^−1^ (phenylpropanoids); 1547 cm^−1^ (shifted from 1523 cm^−1^ in healthy) species (carotenoids)	*A. niger* converts monomeric sugars polymeric carbohydrates
Maize, grain	*Diplodia spp.* [16]	Handheld spectrometer (λ = 1064 nm; P = 200 mW; T = 30 s)	1003–1115 cm^−1^ (monomeric sugars)	1153 cm^−1^ (starch)	Diplodia is associated with a breakdown maize starch into monomeric sugars
*Abutilon hybridum*, leaf	*Abutilon mosaic virus* [29]	Handheld spectrometer (λ = 1064 nm; P = 200 mW; T = 8 s)	1605–1629 (phenylpropanoids); 1440–1460 cm^−1^ (aliphatic)	–	*Abutilon mosaic virus is associated with an increase in* phenylpropanoids in *Abutilon hybridum*
Tomatoes, leaf	Tomato yellow leaf curl Sardinia virus (TYCLSV) [45]	Benchtop spectrometer (λ = 780 nm; P = 2mW; T = 5–10 s)	1608 cm^−1^ (phenolic); 1483 cm^−1^ (aliphatic)	1526 cm^−1^ (carotenoids); 1420, 1483 cm^−1^ (aliphatic), 1500, 1608 cm^−1^ (phenolic); 1353 cm^−1^ (unidentified);	Small changes in plant biochemistry
Tomatoes, leaf	Tomato spotted wilt virus (TSWV) [45]	Benchtop spectrometer (λ = 780 nm; P = 2mW; T = 5–10 s)	1608 cm^−1^ (phenolic); 1438 cm^−1^ (aliphatic); 1353 cm^−1^ (unidentified);	1483 cm^−1^ (aliphatic)	Small changes in plant biochemistry
Wheat, leaf	Barley yellow dwarf virus (BYDV) [36]	Handheld spectrometer (λ = 830 nm; P = 495 mW; T = 1 s)	1601–1630 cm^−1^ (phenylpropanoids)	1000, 1115, 1156, 1186, 1218 and 1525 cm^−1^ (carotenoids)	BYDV is associated with an increase in phenylpropanoids and decrease in carotenoids
Wheat, leaf	Wheat streak mosaic virus (WSMV) [36]	Handheld spectrometer (λ = 830 nm; P = 495 mW; T = 1 s)	1601–1630 cm^−1^ (phenylpropanoids)	1000, 1115, 1156, 1186 and 1218 cm^−1^ (carotenoids)	WSMV is associated with an increase in phenylpropanoids and decrease in carotenoids
Potato, tubers	Zebra chip [112]	Handheld spectrometer (λ = 830 nm; P = 495 mW; T = 1 s)	–	1153 (carbohydrates)	Zebra chip is associated with degradation of carbohydrates in tubers
Potato, tubers	Virus Y [112]	Handheld spectrometer (λ = 830 nm; P = 495 mW; T = 1 s)	1153 cm^−1^ (carbohydrates)		Virus Y is associated with an increase in carbohydrates in tubers
Abiotic stresses					
Coleus lime (*Plectranthus scutellarioides*), leaves	Saline, light, drought and cold [26]	Benchtop spectrometer (λ = 532 nm; P = 10 mW; T = 10 s)	620 and 740 cm^−1^ (anthocyanins)	1000 and 1170 cm^−1^ (carotenoids)	Saline, light, drought and cold stresses cause an increase in anthocyanins and a decrease in carotenoids
*Arabidopsis thaliana*, leaves	Nitrogen deficiency [10]	Postable spectrometer (λ = 830 nm; P = 100 mW; T = 10 s)	–	1064 cm^−1^ (nitrate)	1046 cm^–1^ peak intensity correlates with the nitrate content in *Arabidopsis* plants
Rice, leaves	Nitrogen deficiency [8]	Handheld spectrometer (λ = 830 nm; P = 495 mW; T = 1 s)	1600–1630 cm^−1^ (phenylpropanoids)	1115–1218 cm^−1^ (carotenoids)	Nitrogen deficiency is associated with a decrease in carotenoids and increase in phenylpropanoids
Rice, leaves	Phosphorus and potassium deficiencies [8]	Handheld spectrometer λ = 830 nm; P = 495 mW; T = 1 s)	Small changes in 1600–1630 cm^−1^ (phenylpropanoids)	Small changes in 1115–1218 cm^−1^ (carotenoids)	Phosphorus and potassium deficiencies are associated with a decrease in carotenoids and increase in phenylpropanoids
Identification of plant species and their varieties; nutritional analysis					
Poison ivy, leaves	Farber et al. [36]	Handheld spectrometer (λ = 830 nm; P = 495 mW; T = 1 s)	1717 cm^−1^ (carboxyl or ester groups)		1717 cm^−1^ band can be used to identify poison ivy
Peanuts, leaves and seeds	Farber et al. [36]	Handheld spectrometer (λ = 830 nm; P = 495 mW; T = 1 s)	Identification: all bands Nutritional analysis: 1005 cm^−1^ (proteins), 1301 cm^−1^ (carbohydrates), 1443 cm^−1^ (oils), 1606 cm^−1^ (fiber), 1656 cm^−1^ (unsaturated fatty acids), and 1748 cm^−1^ (esters)		Identification of peanut varieties can be achieved though spectroscopic analysis of leaves and seeds with 80% and 95% accuracy, respectively. RS can be used to predict relative concentration of proteins, carbohydrates, oils, fiber, unsaturated fatty acids and esters in peanut seeds
Potato, tubers	Morey et al. [34]	Handheld spectrometer (λ = 830 nm; P = 495 mW; T = 1 s)	Identification: all bands Nutritional analysis: 1126 cm^−1^ (starch), 1527 cm^−1^ (carotenoids), 1600 cm^−1^ (phenylpropanoids), 1660 cm^−1^ (proteins)		Identification of potato varieties can be achieved though spectroscopic analysis of tubers with 77.5% accuracy. RS can be used to predict relative concentration of proteins, carotenoids, starch and phenylpropanoids in potato tubers
Corn, kernels	Krimmer et al. [21]	Handheld spectrometer (λ = 830 nm; P = 495 mW; T = 1 s)	Identification: all bands Nutritional analysis: 479 cm^−1^ (starch), 1527 cm^−1^ (carotenoids), 1600/1632 cm^−1^ (phenylpropanoids), 1000/1660 cm^−1^ (proteins)		Identification of corn varieties can be achieved though spectroscopic analysis of kernels with 95% accuracy. RS can be used to predict relative concentration of proteins, carotenoids, and starch in corn kernels
Citrus, fruits	Feng et al. [74]	Benchtop spectrometer (λ = 514 nm; P = 20 mW; T = 10 s)	All bands		RS can be used to identify citrus fruits
Loquat, fruits	Zhu et al. [47]	Benchtop spectrometer (λ = 532 nm; P = 25 mW; T = 1 s)	1602 cm^−1^ (lignin)		RS can be used to determine fruit ripening
Tomatoes, fruits	Martin et al. [77]	Benchtop spectrometer (λ = 532 nm; P = 46–50 mW; T = 10 s)	1150, 1257 cm^−1^ (carotenoids)		RS can be used to predict tomato ripeness
Mandarin oranges, fruits	Nekvapil et al. [79]	Benchtop spectrometer (λ = 532 nm; P = 200 mW; T = 10 s)	1100–1250, 1527 cm^−1^ (carotenoids)		RS can be used to predict fruit freshness
Wheat, grain	Piot et al. [80]	Benchtop spectrometer (λ = ’red light’; P = 8 mW)	471–485 cm^−1^ (starch), 1065–1140 cm^−1^ (lipids), 1630–1670 cm^−1^ (protein)		RS can be used to probe concentration of starch, lipids and proteins in the grain
Coffee, beans	Keidel et al. [81]	Benchtop spectrometer (λ = 1064 nm; P = 300 mW)	Identification: all bands Kahweol concentration: 1479 and 1567 cm^−1^		RS can be used to predict the geographical origins of coffee beans
Hemp and cannabis	Sanchez et al. [8]	Handheld spectrometer (λ = 830 nm; P = 495 mW; T = 1 s)	Identification: all bands Cannabinoid content: 780, 1295, 1623, and 1666 cm^−1^		RS can be used to identify cannabis varieties and determine concentrations of cannabinoids in the plant

The beam size of the spectrometer is also linked to the intensity of the laser light. For instance, the use of 495 mW of red laser (λ = 830 nm) with 2 mm beam waist causes no thermal/photodegradation of plant leaves. However, the equivalent laser power (λ = 1064 nm) compressed to 25 microns causes instant burning of the same plant leaf. Therefore, in certain instances, it might be more appropriate to indicate power density rather than laser power for the reported studies. This observation also suggests that it is highly important to demonstrate the absence of laser-induced thermal/photodegradation of plant material in the reported experiments [23].

Most of recently developed hand-held spectrometers weigh only 2–5 lb and can work without charging for 5–8 h. This allows for their direct utilization in the field upon spectroscopic analysis of plants. They also require either direct contact with or a close proximity (0.5–1″) to an analyzed plant. This limits RS to a scout-based approach preventing surveillance of large agricultural territories. One can expect that this problem can be overcome with the use of Raman telescopes. It has been previously shown that the use of telescope reflectors for light collection allows for spectroscopic analysis of samples located ~ 160 ft away from the instrument [24, 25]. Such Raman telescopes can be installed either on motorized vehicles or portable towers in the field to achieve continuous automated surveillance of the agricultural territories.

**Fig. 2:**
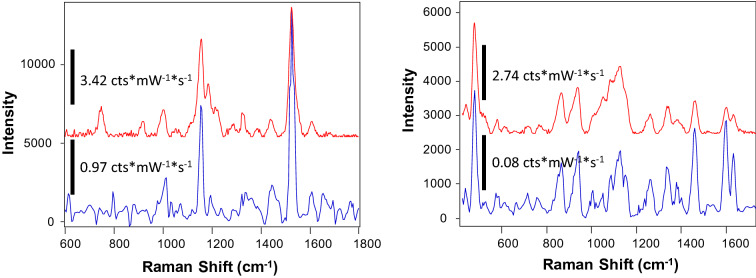
Raman spectra collected form a rose leaf (left) and corn kernel (right) with 830 nm excitation Agilent Resolve (red) (laser power 495 mW, acquisition time 1 s) and 1064 nm excitation Rigaku Progeny (blue) (laser power 350 mW, acquisition time 40 s). Spectral resolution of Agilent Resolve is 15 cm^−1^ and Rigaku Progeny is 8 cm^−1^. Spectral intensity is reported in counts (cts) per milliwatt (mW) per second (s) (cts/mW*s or cts*mW^−1^*s^−1^)

Excitation wavelength is another important instrumental parameter that has to be considered in spectroscopic studies of plants. Our own findings and results reported by other groups [26] show that the use of radiation in the blue and green parts of the electromagnetic spectrum primarily enables visualization of carotenoid signals, Fig. 1C. This can be explained by strong absorption of carotenoids in this part of the electromagnetic spectrum [27]. We also found that lasers with wavelength above 561 and below 700 nm unlikely will suit for structural analysis of life plants due to extremely strong fluorescence of chlorophyll. This fluorescence exponentially decreases at wavelength above 700 nm. Therefore, 785–830 nm laser excitations provide sufficient signal-to-noise spectra of plant leaves, Fig. 1C. For instance, the Ram group recently reported an elegant Raman-based leaf-clip sensor that is based on 830 nm laser excitation [28]. The researchers demonstrated an outstanding potential of this unit for non-invasive diagnostics of the nitrogen deficiency in plants. Several groups demonstrated the possibility of utilization of 1064 nm excitation for Raman-based analysis of maize, wheat, and sorghum grain [15, 16], as well as plant leaves [29]. Although the use of 1064 nm excitation allows for moving even further away from chlorophyll fluorescence, silicon CCDs, which are used for collection of scattered photons in the visible part of the electromagnetic spectrum, have extremely poor photon-to-electron conversion efficiency in Infrared. Therefore, instead of silicon-based CCDs, heterostructure CCDs are used in 1064 nm spectrometers [20]. Such CCDs (typically based on Indium-Gallium-Arsenide (InGaAs)) have much lower photon-to-electron conversion efficiency than silicon CCDs. Therefore, despite no plant fluorescence is present in the IR part of the spectrum, the use of 1064 nm excitation in Raman spectrometers typically results in lower signal-to-noise ratios of collected spectra comparing to the spectra collected using light in the visible part of the electromagnetic spectrum, Fig. 2.

The Raman-based analysis of seeds has its own complications. Phenolic compounds present in seeds may provide strong fluorescence that can obscure Raman readings. Low water content in seeds may also cause photo or thermal degradation of the plant material if high laser power is used. Lastly, the intensity of collected Raman spectra will directly depend on the coloration of the seed. Since Raman is a scattering phenomenon, dark color seeds will scatter less and absorb more photons than light color seeds. This results in overall lower intensity of Raman spectra collected from dark color seeds [21]. In this case, spectra have to be normalized prior to their use for an assessment of the nutrient content based on intensity of corresponding protein or carbohydrate vibrations. Spectral normalization without an internal standard is a challenging task. Kurouski group proposed to use 1460 cm^−1^ band that corresponds to CH_2_ vibration for such normalization [21]. This vibration present in nearly all classes of biological molecules which makes such normalization maximally unbiased.

Electromagnetic radiation in blue-green part of the spectrum does not penetrate as deep in biological tissues as red-near-infrared light. This phenomenon is known as ‘biological window’. Therefore, the wavelength of choice shall be considered accordingly to this optical phenomenon if deeper-lying layers of plant material have to be accessed. It should be noted that this problem can be overcome by spatially-offset Raman spectroscopy (SORS). First introduced by Matousek group [30–32], this technique became broadly used in various research fields ranging from neuroscience [33] to explosive detection [30–32]. Recently, the potential of SORS in digital farming was highlighted by Kurouski group [34]. Morey and co-workers demonstrated that the use of SORS is critically important for non-invasive assessment of nutrient content of potato tubers that otherwise cannot be achieved using normal RS [34].

One may wonder about the extent to which hand-held spectrometers can eliminate the need for the bench-top Raman instruments. We find that bench-top confocal Raman microscopes can be advantageous in two cases: (1) if a very small amount of material is available (below 100 μL); (2) if more fine spectral resolution is required. While the first directly relates to the laser beam size (discussed above), the second aspect is attributed to the spectral resolution of gratings used in hand-held instruments. Most of the commercially available hand-held spectrometers have 8–15 cm^−1^ spectral resolution, whereas the spectral resolution of bench-top instruments reaches 1–2 cm^−1^. Although Raman spectra of plants do not have closely located vibrational bands that may not be resolved with a hand-held instrument (Fig. 1C, 2), 8–15 cm^−1^ spectral resolution might preclude observation of small shifts in vibrational bands. Such shifts provide information about changes in the chemical structure of analyzed specimens. It should be also noted that spectral region in bench-top instruments can be selected by altering a position of the grating. The grating position is not adjustable in the hand-held instruments. These spectrometers typically cover 300–2500 cm^−1^. Although this is sufficient for extensive spectroscopic analysis of plants, high frequency spectral region (2600–4000 cm^−1^), which contains important CH, CH_2_, and OH vibrations, is inaccessible in the hand-held spectrometers.

The research articles discussed in this review will discuss the quantitative nature of RS, which is achieved by the use of advanced statistical analysis, also known as chemometrics. All chemometric methods can be divided into two groups: supervised (methods that require assignments of spectra to groups or classes) and unsupervised (methods that do not require such spectral pre-assignments). There are numerous supervised chemometric methods, including: soft independent modelling of class analogies (SIMCA), partial least squares discriminant analysis (PLS-DA), partial least squares regression (PLSR), and linear discriminant analysis (LDA). Recently reported review by Shashilov and Lednev suggest that all supervised methods perform equivalently well in prediction of the spectral classes [35].

Among all supervised methods, PLS-DA is one of the most commonly used chemometric method in spectroscopy allowing for distinguishing between different groups of spectra [8, 17, 21–23, 36–44]. In each spectral group, the method identifies the most important spectral variables, also known as principal components that can be used to discriminate the assigned groups. PLS-DA outcomes are confusion matrix and principal component spectra. The former demonstrates the accuracy of spectral assignment to the particular group. It also reflects how many spectra were miss-assigned and demonstrates groups to which the model miss-assigned the spectra. The latter can be used to identify vibrational bands that contribute the most to the spectral assignment. Typically, researchers report both confusion matrix and principal component spectra.

In PLS-DA, as well as in other chemometric methods, the prediction accuracy is typically cross-validated using the same set of spectra that were used to develop the model. In some cases, the researchers used a new spectral set to do such validation. In this case, it is described as external validation.

The growing body of evidence demonstrates the use of hand-held Raman spectrometers directly in the field for non-invasive and non-destructive diagnostics of biotic and abiotic stresses [18, 36, 38, 40, 44]. This information can be used for precise, site-specific administration of water, fertilizers, pesticides, and fungicides to a certain field area rather than supplying these valuable resources to the entire field. This allows for fast suppression of pathogen proliferation, as well as reduction of costs associated with these supplies. The impact of Raman-empowered agriculture stretches far beyond diagnostics of biotic and abiotic stresses [8, 18, 26, 29, 36, 39, 40, 42, 45]. Recently reported results show that Raman can be used for non-invasive phenotyping of plant species and their varieties [21, 39]. This allows for development of Raman-based breeding, as RS can be used by farmers and plant biologists working on basic research to reveal information about the species or variety resistance to specific biotic or abiotic stresses [19]. Together with the hand-held nature of RS, this sensitivity of RS can be used for direct in-field screening of plants at early stages of their vegetation. This can drastically reduce time required for plant breeding.

RS also allows for non-invasive assessment of the nutritional values of seeds, which makes it ideal for digital agronomy [46, 47]. It should be noted that RS can be used to unravel the structure of the plant and elucidate plant biochemistry, Table 2. Such information cannot be achieved using conventional imaging techniques such as reflectance spectroscopy, Red, Green and Blue (RGB) and thermography [48–50]. For more detailed discussion of fundamental physical differences between RS and imaging techniques, the reader is advised to visit recently published review by Farber and co-authors [19]. In this review, we critically discuss the most recent progress in Raman-based identification of plant species and their varieties, as well as Raman-based analysis of the nutrition value of seeds. This review aims to attract attention of plant breeders, geneticists, farmers, and engineers to the growing potential of RS in agriculture.

**Table 2:** Vibrational bands and their assignments for spectra collected from plant leaves and seeds

Band (cm-1)	Vibrational mode	Assignment
480	C–C–O and C–C–C Deformations; Related to glycosidic ring skeletal deformations δ(C–C–C) + τ(C–O) Scissoring of C–C–C and out-of-plane bending of C–O	Carbohydrates [51]
520	ν(C–O–C) Glycosidic	Cellulose [52, 53]
747	γ(C–O–H) of COOH	Pectin [54]
849–853	(C_6_–C_5_–O_5_–C_1_–O_1_)	Pectin [55]
917	ν(C–O–C) In plane, symmetric	Cellulose, phenylpropanoids [52]
964–969	δ(CH_2_)	Aliphatics [56, 57]
1000–1005	In-plane CH_3_ rocking of polyene aromatic ring of phenylalanine	Carotenoids [58]; protein
1048	ν(C–O) + ν(C–C) + δ(C–O–H)	Cellulose, phenylpropanoids [52]
1080	ν(C–O) + ν(C–C) + δ(C–O–H)	Carbohydrates [51]
1115–1119	Sym ν(C–O–C), C–O–H bending	Cellulose [52]
1155	C–C Stretching; v(C–O–C), v(C–C) in glycosidic linkages, asymmetric ring breathing	Carotenoids [58],carbohydrates [59]
1185	ν(C–O–H) Next to aromatic ring + σ(CH)	Carotenoids [58]
1218	δ(C–C–H)	Carotenoids [58], xylan [60]
1265	Guaiacyl ring breathing, C–O stretching (aromatic); –C=C–	Phenylpropanoids [61], unsaturated fatty acids [62]
1286	δ(C–C–H)	Aliphatics [56]
1301	δ(C–C–H) + δ(O–C–H) + δ(C–O–H)	Carbohydrates [51, 63]
1327	δCH_2_ Bending	Aliphatics, cellulose, phenylpropanoids [52]
1339	ν(C–O); δ(C–O–H)	Carbohydrates [51]
1387	δCH_2_ Bending	Aliphatics [56]
1443–1446	δ(CH_2_) + δ(CH_3_)	Aliphatics [56]
1515–1535	–C=C– (in plane)	Carotenoids [64–66]
1606–1632	ν(C–C) Aromatic ring + σ(CH)	Phenylpropanoids [67, 68]
1654–1660	–C=C–, C=O Stretching, amide I	Unsaturated fatty acids [62], proteins [64]
1682	COOH	Carboxylic acids [43]
1717–1748	C=O Stretching	Esters, aldehydes, carboxylic acids and ketones [69]

## Raman-based identification of plant species and their varieties

The infamous poison ivy causes allergic reactions due to a mixture of pentadecylcatechols forming urushiol oils [70]. Common symptoms of the victims who come into contact with poison ivy include skin inflammation, uncolored bumps, severe rashes, and blistering [71–73]. It is difficult, however, to differentiate poison ivy from other plants without botanical experience. The Kurouski group helped to overcome this problem by developing non-invasive, non-destructive, confirmatory, and label free approach for detection of poison ivy [38]. Using a hand-held spectrometer, Farber and co-workers found that vibrations of cellulose, carotenoids, phenylpropanoids, pectin, xylan, protein, aliphatic, and carbonyl/ester groups dominated in the Raman spectra collected from poison ivy leaves, Table 2. The researchers also found that Raman spectra collected from other plants exhibited similar bands as the spectrum collected from poison ivy. However, a unique band at 1717 cm^−1^, which can be assigned to carboxyl or ester groups, was not evident in the spectra of other plants. Next, the researchers used partial least square discriminant analysis (PLS-DA) to determine prediction accuracy of different plant species. It has been found that poison ivy could be identified with 100% accuracy. The authors also demonstrated that most of the analyzed plant species could be identified with 100% accuracies. From 10 analyzed plant species, only roses (97.7%), orange (97.7%), grapefruit (97.9%), buckbrush (98.2%), and corn (98.4%) were analyzed with less than 100% accuracy. This work also demonstrated an outstanding sensitivity in RS identification of plant species based on their unique biochemistry.

Confirmatory identification of plant genotypes or varieties can be achieved only by visual recognition of distinct phenotypic appearance (if applicable) or by genotype sequencing. There are many negative aspects with both of these methods. Visual recognition being used to identify genotypes can be difficult and substantial taxonomic expertise is often required. Genotyping by sequencing is time consuming, laborious, and destructive. Recently, Farber and co-authors demonstrated that RS can be used to solve these genotyping identification issues [39]. Chemometric analysis of peanut leaf spectra performed by Farber and co-workers allowed for an accurate identification of both wild and cultivated varieties of peanuts. On average, 80% accuracy was achieved based on collected by Raman spectra from plant leaflets. Peanut variety identification was achieved by spectroscopic identification of vibrational bands that originate from pectin, carotenoids, cellulose, phenylpropanoids, and proteins. The researchers also demonstrated that this approach could be used for prediction of nematode resistance and oleic-linoleic oil (O/L) ratio in peanuts [39]. Using RS analysis, accurate genotype identification could be also achieved by spectroscopic analysis of peanut seeds. Additionally, quantitative information about the concentration of carbohydrates, proteins, fiber, and other nutrients can be determined based on the collected spectra. The same strategy simultaneously enables identification of peanut varieties based on the spectra of their seeds. The results of the scanned seeds showed that RS shows 95% accuracy in identification. It should be noted that both closely related cultivated and wild peanut varieties were used in this study. RS was able to provide accurate identification of both cultivated and wild varieties. Importantly, this accuracy of plant variety identification was achieved using a hand-held instrument.

Independently, Feng and co-workers investigated the accuracy of RS for identification of citrus varieties using RS [74]. The researchers showed that RS coupled to advanced statistical analysis could be used for the confirmatory identification of eight citrus varieties. Further expanding on these results, Zhu and co-workers investigated whether RS could be used for quality assessment of fruit [47]. Optimizing postharvest fruit handling is important to lower quality deterioration. The researchers showed that an increased fruit firmness, known as lignification, could be assessed via RS [47].

## Raman-based assessment of nutritional values of plant seeds

**Fig. 3:**
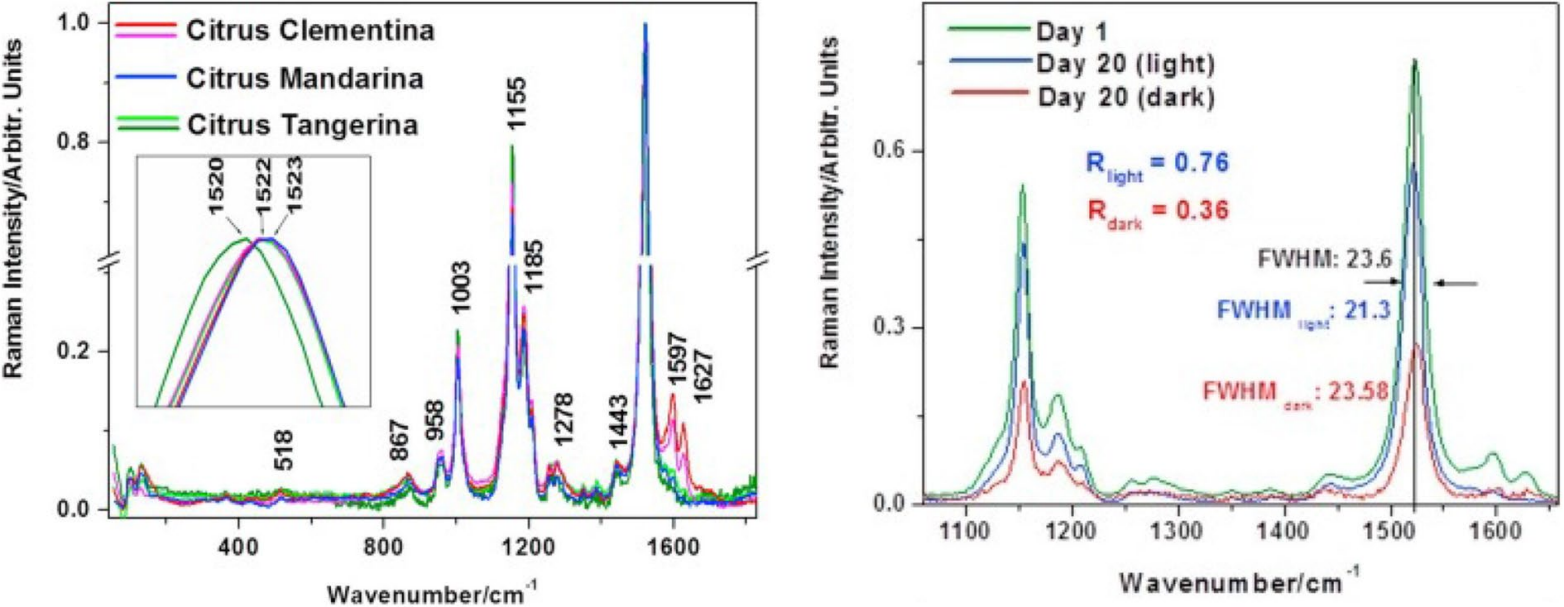
Raman spectra (left) collected from different citrus varieties show distinctly different fruit biochemistry that can be used for citrus variety identification. Primarily differences were found in carotenoids region (1520–1523 cm^−1^) and phenylpropanoid vibrations (1591–1627 cm^−1^). Raman can be also used to determine change in fruit freshness (right) based on changes in vibrational bands of carotenoids. The caption and figure reproduced with permission from Nekvapil et al. [79]

Tomatoes are a broadly cultivated crop that has constantly increasing commercial value. Nikbakht and co-workers used RS to determine the quality of tomatoes [75]. The researchers demonstrated that RS can be used to determine the important quality parameters of intact tomatoes such as soluble solid content (SSC), acidity (pH), and color. Further work was done by Martin and co-workers to expand the use of RS for the assessment of tomato ripening [76]. Carotenoid vibrational bands were used to create a model for tomato ripening. The onset of fruit ripening showed a rise in carotenoid signals after tomatoes were scanned post-harvest. The data collected was used to build a model and delineated ripening stages in tomatoes; the work accurately helps assess fruit quality post-harvest [77]. Zdunek group used Raman microscopy for visualization of the distribution of polysaccharides in cell wall of fruit. The researchers used both single band imaging and multivariate image analysis for the identification and localization of cellulose and pectin in the cell wall in tomatoes [78]. Nekvapil and co-workers further expanded on these findings by investigating RS ability to be used for quality control of fruits [79]. It was shown that RS could be used for fruit freshness. Their results were focused on citrus such as mandarin oranges, tangerines, and clementine (Fig. 3). The results revealed that the freshness of fruit can be determined by the intensity of bands relating to carotenoids in fruit (See Fig. 3). Consumer safety, trust, and satisfaction when purchasing fruits such as citrus can all be improved by using a hand-held Raman spectrometer for quality control [79].

By probing wheat grain with RS, Piot and co-workers followed the evolution of protein content and structure during grain development [80]. It was found that RS not only was able to give information about the structure and composition of grain, but RS was also able to detect molecular species present at low concentrations. One such example would be that of α-helical protein content which was found to increase during grain ripening when kernels harden.

**Fig. 4:**
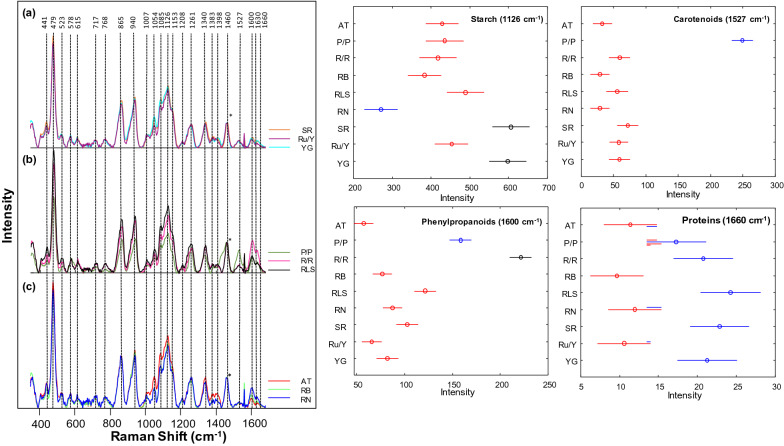
Left: Raman spectra of nine different potato varieties separated into three groups (**a**–**c**) for clarity of visualization. Asterisk (*) denotes 1460 cm^−1^ peak was used to normalize spectra. Right: Means (circles) and confidence intervals for the intensities of the potato spectra at 1126 cm^−1^ (starch), 1527 cm^−1^ (carotenoids), 1600 cm^−1^ (phenylpropanoids) and 1660 cm^−1^ (proteins). ANOVA of starch revealed 3 groups of potato varieties (blue, red and black) with significantly different starch contents. ANOVA of carotenoids revealed 2 groups of potato varieties (blue and red) with significantly different carotenoid contents. ANOVA of phenylpropanoids revealed 3 groups of potato varieties (blue, red, and black) with significantly different phenylpropanoid contents. ANOVA of proteins revealed 2 groups of potato varieties (blue and red) with significantly different protein contents. Multiple colors indicate a member of a group that has overlap between two separate groups. The caption and figure reproduced with permission from Morey et al. [34]

**Fig. 5:**
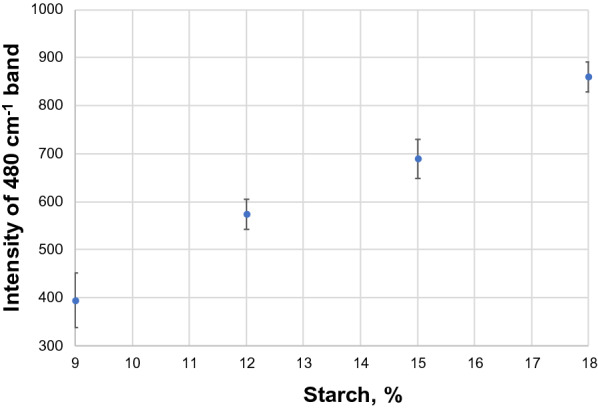
Intensity dependence of 480 cm^−1^ band on the starch content of the sample. Reproduced with permission from Morey et al. [34]

A staple food all over the world, potatoes have a high starch content. Consisting of 83% water and 12% carbohydrates, the remaining 4% of content of potatoes includes proteins, vitamins, and other trace elements [34]. Proportions vary based on both the area of cultivation and potato type. Currently, the chemical methods used to investigate potato starch content are labor consuming, time consuming, indirect, and destructive. A hand-held Raman spectrometer was used by the Kurouski group to assess nutrition value of intact potato tubers [34]. Additionally, nine different potato varieties and origin of cultivation of these potatoes can be determined from the use of RS. The Kurouski group found that the peak intensity varied by potato variety at 479 and 1125 cm^−1^ for starch, 1600 and 1630 cm^−1^ for phenylpropanoid, 1527 cm^−1^ for carotenoid content, and 1660 cm^−1^ for protein content by using offset scans from a hand-held Raman spectrometer, Fig. 4. The researchers were able to determine both area of cultivation and distinguish between potato variety using the differences in relative intensities with 81% to 100% accuracy. The researchers also demonstrated that RS could be used for quantitative assessment of nutritional content of starch in potato tubers. For this, gels with different concentrations of starch were prepared and their spectra were collected. Morey and co-workers showed that the intensity at 480 cm^−1^ in those spectra increased linearly with the increase in the concentration of starch [34]. Using such calibration curves, the researchers were able to accurately determine the absolute rather than relative concentration of starch in potato tubers, Fig. 5.

The researchers showed that Raman spectra collected from the sample with 12% starch (6 g of starch) were statistically different from the spectra collected from 9% (4.5 g of starch) and 15% (7.5 g of starch). Similarly, spectra collected from 15% starch samples were statistically different from the spectra collected from 12 and 18% of starch. Reported standard deviations suggest that the accuracy of quantification of starch lies within 3%. Although such accuracy is expected by potato breeders and farmers, one can expect that more careful standardization may push the prediction accuracy to 1% and below.

Such titration curves are commonly accepted straightforward calibration approach that is used in nearly any analytical methods. However, their direct utilization in RS can be complicated by a laser penetration depth that may vary from sample to sample. Specifically, laser light may penetrate deeper into the starch gel than into the corn kernel. As a result, intensity of 480 cm^−1^ in the former case will be higher than in the latter case upon identical starch concentration in both samples. The problem can be solved if samples with similar laser penetration depths as the desired sample will be used to build the calibration curve. Alternatively, a correction coefficient can be used to adjust for the difference in the sample penetration depth described above.

**Fig. 6:**
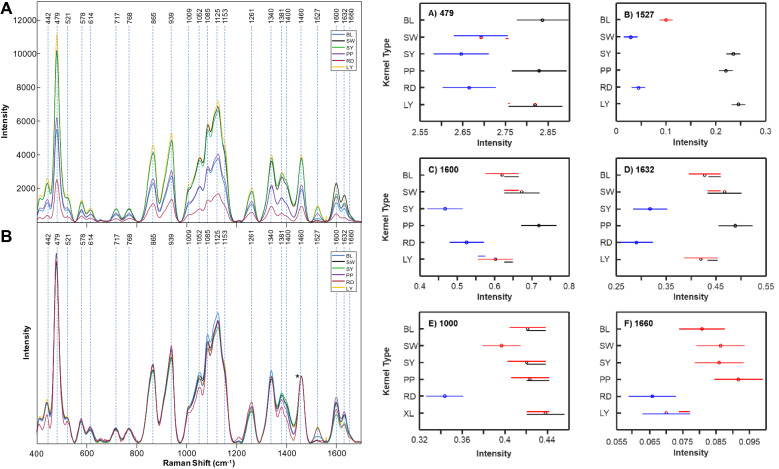
Right: raw (**A**) and normalized (**B**) Raman spectra of BL, SW, SY, PP, RD, and LY maize kernels. The 1458 cm^−1^ peak, which was used for spectral normalization is indicated by an asterisk (*). Left: Means (circles) and confidence intervals for the intensities of the maize kernel spectra, normalized to 1458 cm^−1^, at the indicated wavenumbers. Colors indicate significantly different groups. Multiple colors indicate a member of a group that has overlap between two separate groups. The caption and figure reproduced with permission from Krimmer et al. [21]

One of the most impactful grains in the world is maize, also known as corn. Maize is used as livestock feed, raw material in industry, biofuel, and serves as a staple for human consumption as food and has a commercial impact of more than 50 billion dollars in the United States [16]. Krimmer and co-workers investigate the accuracy of RS for identification of maize varieties and assessment of their nutrient content. It has been found that RS could predict the content of carbohydrates, fibers, carotenoids, and proteins in maize kernels [21], Fig. 6. Using PLS-DA, Krimmer and co-workers also demonstrated that RS could be used to identify maize varieties based on their unique vibrational fingerprints [21].

A question to ask is how accurate is such prediction of nutritional elements. To answer this question, corn material was in parallel examined using near-IR (NIR) spectroscopy and megazyme total starch content assay, methods that commonly used for non-invasive assessment of the starch content of grain. NIR revealed starch variability in the corn from 60.2 to 63.1%, whereas according to the megazyme assay, the amount of starch varied from 54.6 to 59.3%. However, these techniques have substantial internal errors in starch assessment. Specifically, for NIR such error is within ± 5%, whereas for megazyme assay it is within ± 3%. In the light of these facts, two issues became apparent. First, from perspective of both NIR and megazyme assays, the analyzed corn varieties had no statistical difference in the starch content. Second, none of those technique could be used as a reliable calibration metrics for RS.

These findings suggest that RS requires its own calibration approach that has to be developed for highly accurate assessment of the nutritional content of grain.

## Spectroscopic analysis of coffee beans

**Fig. 7:**
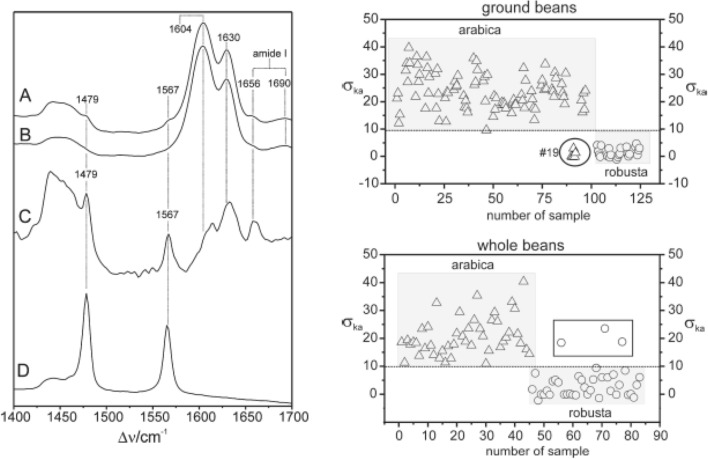
Raman spectra (left) collected from the whole green bean from Arabica (**A**) and from Robusta (**B**). Spectrum (**C**) represents the difference of **A**, **B** to show more clearly the Raman bands of kahweol. The experimental Raman spectrum of neat kahweol is shown in trace (**D**). Results of chemometric analysis of coffee beans (right) demonstrate the possibility of accurate identification of coffee varieties. The caption and figure reproduced with permission from Keidel et al. [81]

Keidel and co-workers used RS to analyze kahweol in whole and ground coffee beans of two different species *Coffea arabica* L. and *Coffea canephora* L. (var. Robusta) grown in Asia, Africa and South America [81]. The researchers found that kahweol could be quantitatively determined with around 3.5% accuracy. It was also shown that spectroscopic signatures of both ground and whole beans could be used to predict the geographical origins of coffee beans, Fig. 7.

Abreu and co-workers further expanded the use of RS for monitoring of coffee quality [82]. Specifically, the researchers questioned whether RS could be used to identify coffee storage conditions and the duration of storage. For this, the researchers collected spectra from coffee beans stored at several different conditions for 0, 3, 6, 9, 12, and 18 months. The researchers found that spectroscopic changes in kahweol could be used to predict quality of coffee and changes that take place in beans upon storage. The same group of researchers also demonstrated that RS could be used for highly accurate differentiation of Arabic coffee genotypes [83]. Coupling RS to principle component analysis, Figueiredo and co-workers were able to differentiate between four genotypes of Arabic coffee: one Mundo Novo line and three Bourbon lines with ~ 80% accuracy.

## Spectroscopic identification of hemp and cannabis varieties

**Fig. 8:**
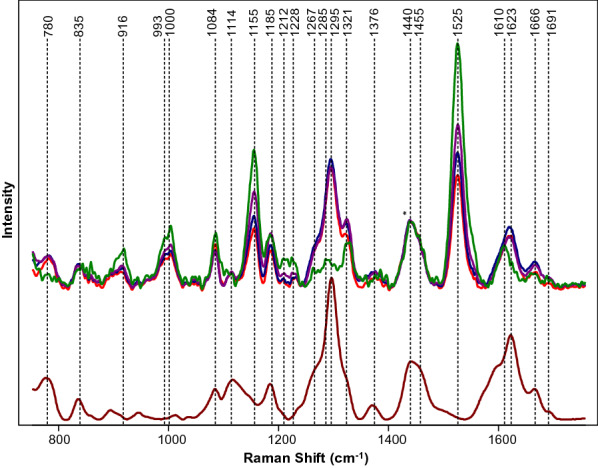
Top: Raman spectra collected form hemp (green), GC (purple), TCC (blue) and TS (red). Bottom: Raman spectrum of THCA extract (maroon). Spectra normalized on CH_2_ vibrations (1440 and 1455 cm^−1^) that are present in nearly all classes in biological molecules (marked by asterisks (*)). The caption and figure reproduced with permission from Sanchez et al. [8]

Since 2900 B.C., hemp has been used to treat pain and a numerous pharmacological effects from an array of cannabinols [84]. With over 100 different cannabinoids present, cannabis plants can have a variety of psychological effects. Delta-9 tetrahydrocannabinol (Δ9-THC), cannabidiol (CBD), and cannabigerol (CBG) are a few of the most studied cannabinoids in which clear psychological effects have been determined [85, 86]. Hemp plants that contain THC in amounts higher than industrial hemp (above 0.3%) is called cannabis. Around 147 million people, which is about 2.5% of the world population, use cannabis [43]. Psychoactive Δ9-THC forms from the oxidation of tetrahydrocannabinolic acid (THCA) that is synthesized by plants. There is a substantial effort from the border control and law enforcement to control illegal trafficking of cannabis as it is the most widely cultivated and trafficked illicit drug in the world. Ideally, growers would want to cultivate cannabis plants with large amounts of CBD and CBG and little to no THC. High performance liquid chromatography is the commonly used method to determine the amount of cannabinoids in plant material [87–90]. However, this method is time consuming, labor consuming, non-portable and destructive. Using orthogonal PLS-DA (OPLS-DA), Sanchez and co-workers recently demonstrated that RS could be used to differentiate with 100% accuracy between hemp, cannabis, and CBD-rich hemp based on spectroscopic analysis of plant buds and leaves [43]. Vibrational bands of cellulose, carotenoids, and lignin were found in the spectrum of hemp using a handheld spectrometer. Key peaks at 780, 1295, 1623, and 1666 cm^−1^ clearly demonstrated a presence of THCA in scanned varieties of hemp and carotenoids had higher intensity in hemp scans relative to cannabis, indicating hemp has a higher carotenoid content compared to cannabis, Fig. 8. It was also found in hemp that higher intensities of cellulose peaks in hemp indicate a higher amount of cellulose in hemp when compared to cannabis. A model was set up to determine if RS could be used to differentiate between hemp and cannabis based on the data collected by Sanchez and co-workers; the results were 100% accuracy. The vibrational band at 1691 cm^−1^ that correlates to the carboxyl group of THCA allows Sanchez and co-workers also to detect THCA in intact growing plants. This key band allowed Sanchez and co-workers to predict the amount of THC in a sample without necessary oxidation of THCA to THC [41]. Another study done by Sanchez and co-workers detected other cannabinoids such as CBD, CBG, cannabigerolic acid (CBGA), and cannabidiolic acid (CBDA) in addition to THCA and THC [41]. Differentiating not only hemp vs cannabis, but also detecting CBD-rich hemp with 100% accuracy is possible thanks to the work done by the Kurouski lab. The extensive study on the six major cannabinoids (THC, THCA, CBD, CBDA, CBG, and CBGA) using RS allows for differentiation between THC/THCA vs CBD/CBDA vs CBG/CBGA and can be used to identify cannabis variety with 97% accuracy [41]. This work also demonstrated the potential of RS to be used upon selection and breeding of hemp and cannabis. Evidence provided by Sanchez and co-workers suggested that RS can be used for on-line monitoring of the plant growth and accumulation of cannabinoids directly in the greenhouse.

Although this work demonstrates that RS can be used for highly accurate differentiation between industrial hemp, cannabis and CBD-rich hemp, it is important to examine the accuracy of determination of the cannabinoid content in the plant. Currently, this accuracy remains unclear. The authors infer that additional work is required to calibrate RS for quantitative and accurate identification of cannabinoids in the plant material.

## Future perspectives

RS is a well-established analytical technique that demonstrated enormous potential in numerous research fields ranging from food chemistry [51] and electrochemistry [91] to forensics [92, 93] and materials science [94] since its discovery in 1928 by C.V. Raman. However, RS’s potential in agriculture and precision farming was largely overlooked for decades. Research results reported by several groups during the last five years demonstrated enormous potential of this technique for: (1) detection and identification of plant diseases [15, 16, 18, 22, 28, 36, 40, 42, 44, 45]; (2) diagnostics of abiotic stresses [8, 26, 28, 95]; (3) spectroscopic identification of plants and elucidation of their phenomics [21, 38, 39, 41, 43], as well as determination of nutritional values of plants and seeds [21, 39]. Constantly growing interest to RS originates from its non-invasive and non-destructive nature that eliminates the need of sampling and sample transportation for the discussed above purposes. RS is also a chemical-free approach. This reduces the cost associated with sample preparation and analyses themselves. Lastly, RS became portable. This allows for its utilization directly in the field, grain elevator, UAV or a combine. Describing all those advantages, it is important to discuss limitations of RS. One of the strongest is the cost of equipment. Although the cost of hand-held Raman spectrometers is comparable to hand-held Infrared spectrometers and a set of equipment required for conventional or real-time PCR, it is largely unaffordable by an average farmer. Therefore, it is highly likely RS would be implemented as a service in agriculture that a farmer may order to investigate the field.

An enhancement of the importance of RS in agriculture will likely to come with required technological advances. First, currently available hand-held spectrometers, although can be used directly in the field, require direct contact with the analyzed sample. Technological solution of this problem will enable the use of RS on UAVs. It is also important to fully understand ideal excitation wavelength for plant sensing, as well as continue miniaturizing and lowering the cost of spectrometers. A large step in broad recognition of RS by farmers and plant breeders is in direct use of this technique in the field. Only a few studies reported to date used RS for direct analysis of plants in the field [18, 22]. Once this practice will become the routine of research studies—the recognition of RS will tremendously increase. It should be also noted that one of the biggest challenges for RS to address in such experiments is diagnostics of several diseases in the same plant, or simultaneous diagnostics of biotic and abiotic stresses.

It should be noted that RS can be further empowered by its coupling to already established imaging [48, 50] and molecular techniques [96–98]. For instance, quick surveillance of the large field territories by UAVs or airplanes equipped with RGB and thermography sensors can be used to navigate RS to the ‘danger’ areas [48, 49, 99]. Such UAV-guided approach can save enormous resources in diagnostics of biotic and abiotic stresses in large agricultural territories. Also, in the light of numerous diseases simultaneously present on a plant, RS can be considered as ‘fast screening’ approach that may be used for rapid screening of plants. If more sophisticated or accurate analysis is needed, molecular methods of analysis such as PCR, qPCR or ELISA can be used [96–98, 100]. In terms of nutritional value assessment, RS goes toe-by-toe with IR-based technologies, which are currently commercialized. In our recent work, we showed that RS- and IR-based technologies are comparable for assessment of nutritional values or grain. RS becomes superior only for wet samples, such as potato tubers [21].

Although this review is focused on RS, technological advances in plant biology and agriculture stretch far beyond this technique. They include an emerging class of sensors that are based on boron-doped diamonds (BDD) [101], single-walled carbon nanotubes (SWNT) [102], and quantum dots [103]. These nano-sensors are capable of probing bioelectric potential changes in plants. This allows for on-line monitoring of temperature, light intensity, and humidity in various plant species [101, 104]. For instance, Strano group demonstrated an outstanding sensitivity of SWNT for detection of stress-induced hydrogen peroxide (H_2_O_2_) signaling waves in seven different plant species [102]. Although the characteristics of the H_2_O_2_ waves appeared to be different across species these responses were specific for the stresses that plants experienced. Such sensors can be also used for detection of volatile organic compounds (VOC), such as ethylene, at parts per billion range [105]. These excellent studies show that significant improvement in understanding of plant genomics and metabolomics can be achieved by development of innovative sensing approaches.

The sensitivity of RS can be amplified by electromagnetic enhancement provided by metal nanostructures upon their illumination by electromagnetic rotation. This phenomenon, first explained by Van Duyne [106], and later determined by Schatz and Moskovitz [107, 108], is known as surface-enhanced Raman spectroscopy (SERS). SERS provides for 10^6^–10^8^ enhancement of Raman scattering enabling single molecule sensitivity. This extremely high sensitivity of SERS can be used to detect plant metabolites present in low concentrations. For instance, Lee et al. used SERS to quantify aflatoxin, a metabolite produced by *Aspergillus flavus*, in corn at a concentration range of 0−1206 μg/kg [109]. SERS was also used to detect turnip yellow mosaic virus (TYMV) in Chinese cabbage leaves [110], as well as mycelia, microconidia, and macroconidia of *Fusarium oxysporum f.* sp. cubense,fungus that infects banana causing Fusarium wilt of banana [111]. These examples suggest that SERS can be a good alternative to RS if additional sensitivity is required for diagnostics of the pathogens.

## Conclusions

This review shows the potential of Raman spectroscopy for digital farming, including timely diagnostics of biotic and abiotic stresses, as well as identification of plants and assessment of plant resistance to certain pathogens such as nematodes. We also discussed how RS can be used to enable digital breeding for drought-resistant peanut lines. We show that high sensitivity possessed by RS has far reaching implications in both plant breeding, botany, and plant pathology. Lastly, we critically review recent research reports that demonstrate the use of RS for determination of nutritional values of peanut seeds and potato tubers. Portability of RS together with its non-invasive and non-destructive nature enhances interest of plant breeders, farmers, basic plant biologists, and pathologists to this emerging analytical technique.

## Data Availability

Not applicable.
